# Peruvian children toothbrushing during the COVID-19 pandemic

**DOI:** 10.12688/f1000research.122504.1

**Published:** 2022-07-07

**Authors:** María Claudia Garcés-Elías, Jorge A. Beltrán, César Eduardo Del Castillo-López, Andrés A. Agudelo-Suárez, Roberto A. León-Manco

**Affiliations:** 1 Facultad de Estomatología, Universidad Peruana Cayetano Heredia, Lima, Peru; 2 Facultad de Odontología, Universidad de Antioquia, Medellín, Colombia

**Keywords:** COVID-19, Toothbrushing, Oral Health, Oral Hygiene, Peru

## Abstract

**Background: **Toothbrushing is a convenient, inexpensive, widespread and culturally accepted method, resulting in an ideal public health outcome. This study aimed to determine the impact of the COVID-19 pandemic on toothbrushing in Peruvian children.

**Methods: **This was a cross-sectional study conducted using a database of children aged 0 to 11 years, with a final sample of 39,124 participants, 15,974 in 2019 (62.03%) and 7088 in 2020 (55.54%). General toothbrushing, daily toothbrushing and minimum frequency of two times a day were dependent variables; the year was considered as the independent variable. In addition, other covariates such as geographical landscape, area of residence, place of residence, altitude, wealth index, health insurance cover, sex and age. Descriptive, bivariate and multivariate statistical analyses were applied.

**Results: **General toothbrushing was 96.19% (n=51 013), daily toothbrushing was 87.47% (n=42 246) and minimum toothbrushing two times a day was 84.53% (n=33 957). In multivariate form, the year presented a negative association with daily toothbrushing (RPa: 0.97; CI95%: 0.96-0.98; p<0.001) and minimum toothbrushing two times a day (RPa: 0.97; CI95%: 0.95-0.98; p<0.001), adjusted for the previously associated co-variables.

**Conclusions: **The year 2020 of the COVID-19 pandemic negatively impacted daily toothbrushing and minimum twice-daily toothbrushing of Peruvian children.

## Introduction

The World Health Organization (WHO) states that diseases of the oral cavity are associated with certain risk factors, including inadequate oral hygiene, which is a practice mediated by the social and commercial determinants of health, and which vary depending on each population. ^1^ It is also established that this healthy habit should be adopted before the first year of life, which would strengthen its continuity over time; it is also recommended that this practice should be done at least twice a day together with the use of fluoride toothpaste, which is an effective measure for the prevention of early childhood caries. It is important to mention that toothbrushing is a convenient, economical, widespread and culturally accepted method, making it an ideal measure for public health. ^2^^,^^3^

On the other hand, the COVID-19 pandemic has caused substantial changes in governments and their populations, which were established to control the spread of the virus and its possible harmful effects on the health of individuals, in addition to avoiding the collapse of health systems. ^4^ Consequently, many of these drastic measures, such as social immobilization, quarantines and cessation of economic activities, had adverse effects on the health and welfare of communities, especially in vulnerable population groups, such as children and adolescents, in whom sociodemographic factors such as age and economic status, in addition to their state of health and special needs, have mediated the magnitude of the impact that the health emergency has had on their daily lives. ^5^ Likewise, these changes in lifestyles could have had repercussions on the exercise of habits that would influence the oral health of children, such as an adequate sleep routine, an increase in the frequency of consumption of foods and beverages with high sugar content and a reduction in the frequency of tooth brushing. ^6^

In Peru, previous reports have documented low adherence to the practice of toothbrushing, especially in individuals under five years of age belonging to families with low income and whose parents had the lowest level of education. In addition, it has been observed that the practice of this habit has been increasing in recent years, despite the fact that, in sectors such as the highlands of the country, there are still differences in its practice. ^7^^,^^8^ However, there are few studies that expose this problem, highlighting the need for its analysis, especially in scenarios as complex as the one that occurred due to the COVID-19 pandemic. In this sense, the purpose of the present investigation was to determine the impact of the COVID-19 pandemic on tooth brushing in Peruvian children.

## Methods

A cross-sectional study was carried out using the databases obtained from the Demographic and Family Health Survey (ENDES) for the years 2019 and 2020, developed by the National Institute of Statistics and Informatics of Peru (INEI); the survey was applied only once per year, through home interviews. The ENDES has a stratified two-stage cluster sample design representative at the national and regional levels, as well as according to rural and urban areas. It also includes information on general toothbrushing, daily toothbrushing and toothbrushing at least twice a day in children from 0 to 11 years of age. In relation to the periods to be evaluated, the year 2019 considered a sample size of 36760 households; while for 2020, the sample size consisted of 37390 households. The primary databases contained a total of 167560 participants for 2019, and for 2020 a total of 177414; however, for this analysis only the records of those who answered the question on general toothbrushing, daily toothbrushing and toothbrushing at least twice a day were considered, establishing a final sample of 56816 subjects, 38203 for 2019 (67.24%) and 18613 for 2020 (32.76%). ^9^

On the other hand, general toothbrushing, daily toothbrushing and toothbrushing at least twice a day were considered as dependent variables, while the year was taken as an independent variable, classified as 2019 and 2020, highlighting that the latter included the period of the COVID-19 pandemic in Peru. Additionally, other covariates were considered within the study, such as the geographical landscape, classified into Metropolitan Lima (capital of the country), rest of the coast, highlands and jungle; area of residence, classified into urban and rural; place of residence organized into capital, city, town and countryside; altitude defined as less than 2,500 meters above mean sea level (MAMSL) and from 2,500 MAMSL and more; in addition, a Wealth Index, a variable defined by the willingness of each household to use and consume goods and services. Then, through the method used in the Demographic and Health Survey Program of the United States, a score was assigned to each household, as well as to each of its inhabitants, which served to categorize each dwelling, following a hierarchy that goes from quintile one to quintile five (from the poorest to the richest). ^10^^,^^11^ Likewise, the holding of health insurance was considered, considering that within Peru there are simultaneously public and private insurance institutions. The public sector includes: Integrative Health Insurance (SIS), Peruvian Social Security (EsSalud), Armed Forces Health Insurance and Police Forces Health Insurance; while in the public or private sector, Health Care Provider Companies (EPS) offer additional coverage to that provided by social security and its essential plan. ^12^ On the other hand, the covariables sex and age, classified as 0 to 5 years and 6 to 11 years were also considered. Similarly, these have been analyzed in previously published research. ^7^^,^^8^

The databases were extracted from the INEI's official website, through multiple sources of information, and then unified into a single matrix to be analyzed in STATA v. 17 software. This was done using the complex sample module, since it is a national survey with possible representative estimates.

### Statistical analysis

The statistical analysis began with a descriptive analysis for each of the variables to obtain their absolute and relative frequencies. Subsequently, we continued with a bivariate analysis using a Chi-square test that allowed us to find associations between the variables studied. Next, for multivariate analysis, tests such as Poisson regression were run to obtain crude prevalence ratios (PR) and adjusted prevalence ratios (aPR). It is worth mentioning the svy command was used to determine representative estimates, because the survey design was included in the data analysis, where the sampling patterns were differentiated in the stratum, primary sampling unit and weights. For this research, a confidence level of 95% and a value of p<0.05 was used as an indicator of statistical significance in all tests.

### Ethical considerations

For the development of this study, the researchers used freely available information provided by the INEI which, since it is a secondary analysis of anonymous information, does not require ethical approval.

## Results

General toothbrushing was 96.19% (n=51 013), daily toothbrushing was 87.47% (n=42 246) and minimum toothbrushing two times a day was 84.53% (n=33 957); the sample was mainly associated to metropolitan Lima with 34.20% (n=10 125), 77.53% (n=50 037) from urban areas, 34.20% (n=101 125) from the capital and 78.79% (n=52 378) residing at less than 2 500 MAMSL. According to the Wealth Index, 22.06% (n=19 244) were mainly poor participants, while 75.38% (n=228 594) had health insurance. A total of 74.51% (n=138 395) were male and 62.74% (n=25 194) were between six and 11 years of age ( Table 1).

**Table 1.** Characteristics of the sample of Peruvian children under 12 years old.

Variables		n	%
Year			
	2019	167560	66.32
	2020	177414	33.68
General toothbrushing			
	Yes	51013	96.19
	No	5803	3.81
Daily toothbrushing			
	Yes	42246	87.47
	No	8738	12.53
Toothbrushing at least twice a day			
	Yes	33957	84.53
	No	8289	15.47
Geographical landscape			
	Metropolitan Lima	10125	34.20
	Rest of coast	21041	24.80
	Highlands	27282	25.77
	Jungle	17109	15.23
Area of residence			
	Urban	50037	77.53
	Rural	25520	22.47
Place of residence			
	Capital	10125	34.20
	City	20769	20.55
	Town	19143	22.78
	Countryside	25520	22.47
Altitude			
	Less than 2500 MAMSL	52378	78.79
	From 2500 MAMSL and more	23179	21.21
Wealth index			
	Very poor	21084	20.02
	Poor	19244	22.06
	Medium	15181	21.34
	Rich	11901	19.27
	Very rich	8820	17.31
Health insurance			
	With insurance	228594	75.38
	Without insurance	54545	24.62
Sex			
	Man	138395	74.51
	Woman	144744	25.49
Age			
	From 0 to 5 years old	37431	37.26
	From 6 to 11 years old	25194	62.74

n: Absolute frecuency. %: Relative frequency.

In a bivariate manner, general toothbrushing was associated with geographical landscape, area of residence, place of residence, altitude and age (p<0.05); daily toothbrushing was associated with year, geographical landscape, area of residence, place of residence, altitude, Wealth Index and age (p<0.05); and minimum toothbrushing two times a day was associated with year, geographical landscape, area of residence, place of residence, altitude, Wealth Index, health insurance cover, sex and age (p<0.05) ( Table 2). In multivariate form, the year presented a negative association with daily toothbrushing (RPa: 0.97; 95%CI: 0.96-0.98; p<0.001) and minimum toothbrushing two times a day (RPa: 0.97; 95%CI: 0.95-0.98; p<0.001) adjusted for the previously associated co-variables ( Table 3).

**Table 2.** Toothbrushing according to characteristics of Peruvian children under 12 years old.

Variables		General toothbrushing					Daily toothbrushing					Toothbrushing at least twice a day				
		Yes		No		p *	Yes		No		p *	Yes		No		p *
		n	%	n	%		n	%	n	%		n	%	n	%	
Year																
	2019	34198	96.32	4005	3.68	0.202	28444	86.29	5735	13.71	0.009	23060	85.44	5384	14.56	<0.001
	2020	16815	95.94	1798	4.06		13802	88.05	3003	11.95		10897	82.66	2905	17.34	
Geographical landscape																
	Metropolitan Lima	3903	97.42	172	2.58	<0.001	3488	90.80	412	9.20	<0.001	2972	86.57	516	13.43	<0.001
	Rest of coast	9153	96.11	532	3.89		8081	90.31	1065	9.69		6784	86.22	1297	13.78	
	Highlands	10319	95.41	736	4.59		7719	79.15	2593	20.85		6121	80.72	1598	19.28	
	Jungle	7792	95.08	502	4.92		6878	89.69	910	10.31		5565	83.00	1313	17.00	
Area of residence																
	Urbano	21670	99.09	1091	0.91	0.002	18872	89.46	2780	10.54	<0.001	15580	85.35	3292	14.65	<0.001
	Rural	9497	98.62	851	1.38		7294	80.65	2200	19.35		5862	81.43	1432	18.57	
Place of residence																
	Capital	3903	99.45	172	0.55	<0.001	3488	90.80	412	9.20	<0.001	2972	86.57	516	13.43	<0.001
	City	9193	98.83	446	1.17		8030	89.09	1155	10.91		6541	84.64	1489	15.36	
	Town	8574	98.82	473	1.18		7354	87.90	1213	12.10		6067	84.19	1287	15.81	
	Countryside	9497	98.62	851	1.38		7294	80.65	2200	19.35		5862	81.43	1432	18.57	
Altitude																
	Less than 2500 MAMSL	22434	99.13	1303	0.87	<0.001	19765	90.14	2654	9.86	<0.001	16427	85.66	3338	14.34	<0.001
	From 2500 MAMSL and more	8733	98.47	639	1.53		6401	77.74	2326	22.26		5015	79.77	1386	20.23	
Wealth index																
	Very poor	7965	98.78	760	1.22	0.179	6104	80.62	1861	19.38	<0.001	4899	82.15	1205	17.85	<0.001
	Poor	7852	99.01	475	0.99		6546	84.72	1303	15.28		5299	83.03	1247	16.97	
	Medium	5982	99.22	307	0.78		5182	88.87	792	11.13		4266	84.78	916	15.22	
	Rich	4468	98.94	182	1.06		3952	90.59	514	9.41		3278	84.87	674	15.13	
	Very rich	3239	98.81	121	1.19		2980	94.11	256	5.89		2595	89.70	385	10.30	
Health insurance																
	With insurance	40792	99.01	4710	0.99	0.403	33679	87.60	7091	12.40	0.472	27262	85.33	6417	14.67	<0.001
	Without insurance	10221	98.90	1093	1.10		8567	87.06	1647	12.94		6695	82.03	1872	17.97	
Sex																
	Man	28374	98.95	2621	1.05	0.280	23686	87.46	4673	12.54	0.990	19323	85.12	4363	14.88	0.013
	Woman	22639	99.09	3182	0.91		18560	87.47	4065	12.53		14634	82.83	3926	17.17	
Age																
	From 0 to 5 years old	26283	97.44	5339	2.56	<0.001	20764	83.00	5502	17.00	<0.001	15963	81.01	4801	18.99	<0.001
	From 6 to 11 years old	24730	99.74	464	0.26		21482	89.66	3236	10.34		17994	86.12	3488	13.88	

n: Absolute frequency. %: Relative frequency. p: Statistical significance.

* Chi-square test.

**Table 3.** Association between toothbrushing and year of the COVID-19 pandemic in children under 12 years old in Peru.

Variables		General toothbrushing						Daily toothbrushing						Toothbrushing at least twice a day					
		PR	95%CI	p	aaPR	95%CI	p	PR	95%CI	p	abPR	95%CI	p	PR	95%CI	p	acPR	95%CI	p
Year																			
	2019	Ref.			Ref.			Ref.			Ref.			Ref.			Ref.		
	2020	1.00	0.99-1.00	0.212	0.99	0.99-1.00	0.654	0.98	0.97-0.99	0.010	0.97	0.96-0.98	<0.001	0.97	0.95-0.99	<0.001	0.97	0.95-0.98	<0.001
Geographical landscape																			
	Metropolitan Lima	Ref.						Ref.						Ref.					
	Rest of coast	0.99	0.95-1.02	0.484	-	-	-	0.99	0.95-1.03	0.549	-	-	-	0.99	0.94-1.03	0.499	-	-	-
	Highlands	0.97	0.94-1.01	0.170	-	-	-	0.84	0.80-0.87	<0.001	-	-	-	0.94	0.89-0.97	0.001	-	-	-
	Jungle	0.98	0.94-1.02	0.325	-	-	-	0.99	0.95-1.03	0.544	-	-	-	0.95	0.91-0.99	0.023	-	-	-
Area of residence																			
	Urban	Ref.						Ref.						Ref.					
	Rural	0.96	0.95-0.97	<0.001	-	-	-	0.90	0.89-0.92	<0.001	-	-	-	0.95	0.94-0.97	<0.001	-	-	-
Place of residence																			
	Capital	Ref.						Ref.						Ref.					
	City	1.00	0.99-1.00	0.202	-	-	-	0.98	0.96-1.00	0.057	-	-	-	0.98	0.95-1.00	0.064	-	-	-
	Town	0.99	0.98-0.99	0.010	-	-	-	0.97	0.95-0.99	0.001	-	-	-	0.97	0.95-0.99	0.022	-	-	-
	Countryside	0.96	0.95-0.97	<0.001	-	-	-	0.89	0.87-0.91	<0.001	-	-	-	0.94	0.92-0.96	<0.001	-	-	-
Altitude																			
	Less than 2500 MAMSL	Ref.						Ref.						Ref.					
	From 2500 MAMSL and more	0.99	0.98-0.99	0.001				0.86	0.85-0.88	<0.001				0.93	0.91-0.95	<0.001			
Wealth index																			
	Very poor	Ref.						Ref.						Ref.					
	Poor	1.03	1.00-1.07	0.050	-	-	-	1.09	1.05-1.13	<0.001	-	-	-	1.01	0.97-1.05	0.665	-	-	-
	Medium	1.04	1.01-1.08	0.016	-	-	-	1.13	1.09-1.17	<0.001	-	-	-	1.03	0.98-1.07	0.225	-	-	-
	Rich	1.05	1.01-1.09	0.006	-	-	-	1.15	1.11-1.20	<0.001	-	-	-	1.03	0.99-1.08	0.145	-	-	-
	Very rich	1.06	1.01-1.10	0.009	-	-	-	1.20	1.15-1.26	<0.001	-	-	-	1.09	1.03-1.14	0.001	-	-	-
Health insurance																			
	Without insurance	Ref.						Ref.						Ref.					
	With insurance	1.01	0.99-1.01	0.072	-	-	-	1.01	0.99-1.02	0.478	-	-	-	1.04	1.02-1.06	0.001	-	-	-
Sex																			
	Man	Ref.						Ref.						Ref.					
	Woman	1.00	0.99-1.01	0.297	-	-	-	1.00	0.98-1.02	0.990	-	-	-	0.97	0.95-0.99	0.018	-	-	-
Age																			
	From 0 to 5 years old	Ref.						Ref.						Ref.					
	From 6 to 11 years old	1.09	1.08-1.10	<0.001	-	-	-	1.08	1.06-1.10	<0.001	-	-	-	1.06	1.04-1.08	<0.001	-	-	-

PR: Prevalence ratio; aPR: Ajusted prevalence ratio; 95% CI: 95% confidence intervals; p: Statistical significance.

a: Adjusted for Geographical landscape, Area of residence, Place of residence, Altitude and Age.

b: Adjusted for Geographical landscape, Area of residence, Place of residence, Altitude, Wealth Index and Age.

c: Adjusted for Geographical landscape, Area of residence, Place of residence, Altitude, Wealth Index, Health Insurance, Sex and Age.

## Discussion

Toothbrushing stands out as one of the main protective strategies for oral health, especially when it is done with fluoride toothpaste; therefore, it is essential to guarantee the practice of this habit in population groups with a higher risk of dental caries, such as children. In this regard, there are factors associated with the frequency of this practice, such as the frequency of brushing by parents and caregivers, as well as having received help from others; in addition, barriers have been reported such as lack of time to carry it out and little cooperation from the child. This shows that healthy behaviors such as tooth brushing in children, are linked to the parents' own practices and family support during its realization. ^13^^,^^14^

Among the findings of this research, it was observed that the year of the COVID-19 pandemic outbreak negatively impacted daily toothbrushing and its practice frequency of at least twice a day. Coincidentally, some studies evaluated whether the changes caused by the COVID-19 pandemic would have affected oral health habits, finding a reduction in the frequency of toothbrushing. Gotler *et al.* reported that approximately a quarter of the subjects studied reduced the frequency of this practice both during the day and at night; they also observed that this phenomenon was reported mainly in older children. ^6^ Additionally, in another study applied to parents in the same context, the proportion of children whose oral health had worsened was remarkable, despite their brushing practices. Furthermore, they highlighted that, in order to maintain adequate oral hygiene, there are mediating factors such as the degree of awareness, socioeconomic level, access to health services, among others. ^15^

Likewise, another study in Brazil evaluated whether the modification of sleeping habits would influence the oral hygiene of children during the period of confinement, observing that the emergence of changes in the family routine of parents or caregivers would be relevant in the oral hygiene of the child, especially in the younger pediatric population, who depend on adults to perform tooth brushing. ^16^ On the other hand, Folayan *et al.* reported that in the COVID-19 pandemic, one tenth of the adult individuals evaluated reported having reduced the regularity with which they brushed their teeth; likewise, the interruption of this healthy habit would be linked to the development of disorders such as anxiety and decreased psychological well-being. ^17^ From the aforementioned, it can be derived that the modification of such an important daily routine due to such complex scenarios as a health emergency would influence the deterioration of oral hygiene maintenance.

Regarding general brushing, this research determined that there is an association between this type of practice and the geographical characteristics surrounding the children, such as geographical landscape, area, place of residence and altitude. In 2017, Hernández-Vásquez *et al*. concluded that a considerable fraction of Peruvian children under 12 years of age did not practice toothbrushing, especially those settled in the highlands and urban sectors of Peru; likewise the absence of this habit was observed with greater predominance in those wiofth five years of age or younger. ^7^ A study applied in the United Arab Emirates showed that children whose day-care centers were located in rural areas had a greater experience of caries and a higher amount of visible plaque compared to other geographical areas; in addition, the same publication established infrequent tooth brushing as a factor associated with the high prevalence of dental caries in that community, ^18^ which could explain the health conditions of these children.

In addition, it is noted that age is also associated with general toothbrushing, in line with previous studies such as that of Sun *et al.* in China, where it was found that slightly less than half of the participating children brushed their teeth less than once a day, showing the great risk to which children in China under five years of age are exposed; there was also a marked relationship between the age of initiation of toothbrushing and the experience of childhood caries. ^19^ However, as an indicator, general toothbrushing is generic, due to it only evaluating the performance or not of this practice, without considering its regularity.

On the other hand, in addition to the characteristics previously mentioned, the Wealth Index also presents statistical differences when evaluating daily toothbrushing; it was observed that the greater the purchasing power, the greater the frequency of this type of brushing. A study in Brazil showed that, for the most part, the frequency of brushing in children was twice a day; however, those who brushed only once during this period predominantly belonged to families with a low socioeconomic level. Furthermore, the researchers ratified the fact that compliance with this preventive habit depended on the commitment and collaboration of parents or caregivers. ^20^ In addition, Chen *et al.* reported an evident positive gradient between the frequency of child toothbrushing and the educational levels of the parents, observing that the children of parents with a higher level of education had a higher propensity to brush twice a day or more, ^21^ implying the relationship between a higher level of education and economic stability.

Toothbrushing at least twice a day together with toothpaste with fluoride levels equal to or higher than 1000 parts per million, turns out to be an adequate preventive practice as established in the Peruvian regulations, through the Clinical Practice Guidelines for the prevention, diagnosis and treatment of dental caries in girls and boys, in force since 2017. ^22^ Within this research, it was mentioned that exercising this habit twice a day presents association with all the variables analyzed, such as geographical landscape, area and place of residence, altitude, Wealth Index, health insurance cover, sex and age. In this regard, a previous study using the ENDES determined that between 2013 and 2018 in Peru, there was a progressive trend of adequate brushing frequency, defined as the exercise of tooth brushing from twice a day to more. However, in the rural communities of the country, the percentage of children who exercise this practice is significantly lower, which can also be observed in the Sierra region. ^8^

Likewise, Soltani *et al.* documented that by 2014, toothbrushing frequency in Iranian children aged four to six years was low; in addition, it was associated with socioeconomic and demographic factors, and access to health services, ^23^ a similar result to those presented in this study. In this sense, it was noted that the evaluation of the habit of brushing two or more times presents a greater number of associated factors, and it is observed that the better the living conditions, the higher the frequency of tooth brushing. In this sense, it is understood that national surveys provide essential information on the performance of hygiene practices necessary for oral health care; however, there are indicators that show greater precision and degree of compliance with the expected result, such as the one that evaluates the performance of the practice as established by the health authority, and not only analyzes without considering the continuity and precision of the preventive habit.

Certain limitations were generated by the study, where the nature of the design should be considered: being a cross-sectional study, does not allow establishing causal relationships to the phenomenon studied. In addition, since the information was obtained from secondary sources, inaccuracies in the results are a possibility, due to possible errors at the time of collection, as well as the emergence of recall bias, due to self-reporting by the participants. Despite the above, the ENDES continues to be an important source of information on the oral health situation with national representativeness.

It is important to consider toothbrushing as a fundamental hygiene habit for the prevention of diseases of the oral cavity, so the establishment and maintenance of this habit should begin as early as possible, in order to promote optimal conditions of health and quality of life that are sustainable over time. Despite this, in Peru there have been conditions and social characteristics that have established differences in the practice of this preventive habit. However, as a result of the COVID-19 pandemic and its consequent provisions, the oral hygiene practices of Peruvian children under 11 years of age were affected, especially in those facing conditions of greater vulnerability, which already existed prior to the health emergency, but which during this complex situation have worsened and extended to a greater part of the population. It is necessary to address this problem from a multidisciplinary perspective, involving the participation of health professionals, parents, teachers and other stakeholders. Furthermore, the political decision-makers must ensure compliance with their programs and interventions in oral health and monitor the results achieved, paying special attention to indicators based on scientific evidence, which will reveal with certainty whether the oral health of Peruvian children is at risk.

In this sense, the year 2020 of the COVID-19 pandemic negatively impacted daily toothbrushing and minimum twice daily toothbrushing of Peruvian children; the associated factors being geographical landscape, area of residence, place of residence, altitude, health insurance coverage, Wealth Index, sex and age.

## Data availability

The data analyzed are freely accessible and provided by the National Institute of Statistics and Informatics of Perú, for 2019 ( https://www.datosabiertos.gob.pe/dataset/encuesta-nacional-demograf%C3%ADa-y-salud-familiar-endes-2019-instituto-nacional-de-estad%C3%ADstica-4/) and 2020 ( https://www.datosabiertos.gob.pe/dataset/encuesta-demográfica-y-de-salud-familiar-endes-2020-instituto-nacional-de-estad%C3%ADstica-e-2).
