# Supplementary material for: Peruvian children toothbrushing during the COVID-19 pandemic
Source: F1000Res. 2022 Nov 8;11:760. Originally published 2022 Jul 7. [Version 2] doi: 10.12688/f1000research.122504.2 (PMC9692047; doi:10.12688/f1000research.122504.2)
Supplement: Peer review history

# Peer review: Reviewer response for version 2

**DOI:** 10.5256/f1000research.140775.r155297

**Published:** 2022-11-24

**Authors:** Iris Lucia Espinoza Santander

**Affiliations:** 1 Center for Epidemiology and Surveillance of Oral Diseases (CESOD) and Department of Pathology and Oral Medicine, Faculty of Dentistry, University of Chile, Santiago, Chile

I consider that the authors have made the requested clarifications, improving the methodology and discussion sections. The article shows an interesting result and recommends its indexing.

 Delete paragraph in discussion: *"Certain limitations were generated by the study, where the nature of the design should be considered: being a cross-sectional study, does not allow establishing causal relationships to the phenomenon studied. In addition, since the information was obtained from secondary sources, inaccuracies in the results are a possibility, due to possible errors at the time of collection, as well as the emergence of recall bias, due to self-reporting by the participants. Despite the above, the ENDES continues to be an important source of information on the oral health situation with national representativeness",* due to these aspects being included in the second paragraph of the discussion in the new version.

Is the work clearly and accurately presented and does it cite the current literature?

Yes

If applicable, is the statistical analysis and its interpretation appropriate?

I cannot comment. A qualified statistician is required.

Are all the source data underlying the results available to ensure full reproducibility?

Yes

Is the study design appropriate and is the work technically sound?

Partly

Are the conclusions drawn adequately supported by the results?

Yes

Are sufficient details of methods and analysis provided to allow replication by others?

Partly

Reviewer Expertise:

Oral Pathology and Inequalities in Oral Disease

I confirm that I have read this submission and believe that I have an appropriate level of expertise to confirm that it is of an acceptable scientific standard.

# Peer review: Reviewer response for version 2

**DOI:** 10.5256/f1000research.140775.r155296

**Published:** 2022-11-15

**Authors:** Gino Montenegro Martínez

**Affiliations:** 1 Graduate School, CES University, Medellín, Colombia

The authors complete all the suggestions. The article provides essential information about sanitary measures for covid-19 in oral health.

Is the work clearly and accurately presented and does it cite the current literature?

Yes

If applicable, is the statistical analysis and its interpretation appropriate?

Yes

Are all the source data underlying the results available to ensure full reproducibility?

Yes

Is the study design appropriate and is the work technically sound?

Partly

Are the conclusions drawn adequately supported by the results?

Partly

Are sufficient details of methods and analysis provided to allow replication by others?

Yes

Reviewer Expertise:

Public health

I confirm that I have read this submission and believe that I have an appropriate level of expertise to confirm that it is of an acceptable scientific standard.

# Peer review: Reviewer response for version 1

**DOI:** 10.5256/f1000research.134498.r150600

**Published:** 2022-10-31

**Authors:** Gino Montenegro Martínez

**Affiliations:** 1 Graduate School, CES University, Medellín, Colombia

**Introduction**

 The pandemic for covid-19 has relevant implications for population health; one of them relates to oral health. This article addresses a pertinent topic for dentistry; it is well-written and articulates the research problem very well. 

 It is necessary to introduce other characteristics related to toothbrushing practices in children, supported by literature, to be part of the discussion of the results. In the last phrase of the introduction section, the authors write, “there are few studies that expose this problem,” but there are no references to those studies.

****

**Methods**

 The design is according to the objective of the study. The authors explain the process followed to get the results. It is recommended to define the reference category for statistical procedures.

****

**Results**

 It is well-written; the description of the tables is accurate and highlights relevant results for the study.

****

**Discussion**

****

 The discussion section shows other factors related to toothbrushing practice unavailable in this study because of source information. In this sense, it is essential to consider this limitation in the study conclusions. 

 The discussion section should be oriented to explicate this study's results more than comparing them.

Is the work clearly and accurately presented and does it cite the current literature?

Yes

If applicable, is the statistical analysis and its interpretation appropriate?

Yes

Are all the source data underlying the results available to ensure full reproducibility?

Yes

Is the study design appropriate and is the work technically sound?

Partly

Are the conclusions drawn adequately supported by the results?

Partly

Are sufficient details of methods and analysis provided to allow replication by others?

Yes

Reviewer Expertise:

Public health

I confirm that I have read this submission and believe that I have an appropriate level of expertise to confirm that it is of an acceptable scientific standard, however I have significant reservations, as outlined above.

# Response

**Published:** 2022-11-04

**Authors:** MARIA CLAUDIA GARCES ELIAS

Dear reviewer, we appreciate your suggestions, recommendations and corrections. These were answered below:

 1. Introduction: In the last phrase of the introduction section, the authors write, “there are few studies that expose this problem,” but there are no references to those studies.

 Accepted and the change was made.

 2. Methods: It is recommended to define the reference category for statistical procedures.

 Accepted and the change was made.

 3. Discussion: The discussion section shows other factors related to toothbrushing practice unavailable in this study because of source information. In this sense, it is essential to consider this limitation in the

 study conclusions. 

 Accepted and included within the study limitations.

 4. The discussion section should be oriented to explicate this study's results more than comparing

 them.

 It is accepted and in the main findings paragraph information is added and an explanation of the findings is written.

# Peer review: Reviewer response for version 1

**DOI:** 10.5256/f1000research.134498.r145020

**Published:** 2022-08-22

**Authors:** Iris Lucia Espinoza Santander

**Affiliations:** 1 Center for Epidemiology and Surveillance of Oral Diseases (CESOD) and Department of Pathology and Oral Medicine, Faculty of Dentistry, University of Chile, Santiago, Chile

This is a study with the aim to determine the impact of the COVID-19 pandemic on toothbrushing (general toothbrushing, daily toothbrushing and toothbrushing at least twice a day) in Peruvian children from 0 to 11 years of age. The study was carried out using the databases obtained from the Demographic and Family Health Survey (ENDES) for the years 2019 and 2020, developed by the National Institute of Statistics and Informatics of Peru (INEI).The article is generally well written and structured. However, in my opinion the paper has some shortcomings regarding text structure and minor inconsistencies. Below I have provided remarks:

**Methods**

 The author should explain what is the mean of the variable “general tooth brush”. The authors should write in methods the questions (dependent variables) used in the surveys 2019 and 2020. Were the same questions used in both years? This should be explicitly mentioned. Additionally, the authors should mention the reference categories in General toothbrushing, Daily toothbrushing and Toothbrushing at least twice a day (table 3).

**Discussion**

 The text of the discussion seems a bit long and it does not contain the most appropriate order.

 I suggest considering a reorder the discussion ^1^. The first paragraph of the discussion should briefly reiterate what the study did and what it showed, statement of principal findings. The second paragraph should address the Strengths and weaknesses of the study. The paragraphs which follow should then discuss how the findings support or refute the current literature. The final paragraph should tie it all together – so what? Where next? What are the implications for practice? Unanswered questions and future research.

Is the work clearly and accurately presented and does it cite the current literature?

Yes

If applicable, is the statistical analysis and its interpretation appropriate?

I cannot comment. A qualified statistician is required.

Are all the source data underlying the results available to ensure full reproducibility?

Yes

Is the study design appropriate and is the work technically sound?

Partly

Are the conclusions drawn adequately supported by the results?

Yes

Are sufficient details of methods and analysis provided to allow replication by others?

Partly

Reviewer Expertise:

Oral Pathology and Inequalities in Oral Disease

I confirm that I have read this submission and believe that I have an appropriate level of expertise to confirm that it is of an acceptable scientific standard, however I have significant reservations, as outlined above.

## References

[1] : The case for structuring the discussion of scientific papers. *BMJ* .1999;318(7193) : 10.1136/bmj.318.7193.1224 1224-1225 10.1136/bmj.318.7193.1224 PMC111562510231230

# Response

**Published:** 2022-11-04

**Authors:** MARIA CLAUDIA GARCES ELIAS

Dear reviewer, we appreciate your comments, suggestions and corrections. We will respond to them below:

 1.Explain what is the meaning of the variable “general tooth brush”, write in methods the questions (dependent variables) used in the surveys 2019 and 2020 and mention if they are the same questions used in both years, mention the reference categories in dependent variables.

 We accepted what was suggested and the modifications were made in the indicated sections (materials and methods and Table 3).

 2. Reorder the discussion, the first paragraph of the discussion should briefly reiterate what the study did and what it showed. The second paragraph should address the strengths and weaknesses of the study. The paragraphs which follow should then discuss how the findings support or refute the current literature. The final paragraph should tie it all together – so what? Where next? What are the implications for practice? Unanswered questions and future research.

 Suggestions were accepted and the order of the discussion was modified as recommended by the reviewer. The first and second paragraphs were modified as indicated and the penultimate paragraph, where the final idea is rounded off and recommendations are made, was maintained.
